# The efficacy and safety of PD-1 inhibitors combined with ^125^I seed implantation in treating extrahepatic metastasis of hepatocellular carcinoma

**DOI:** 10.3389/fmed.2025.1693447

**Published:** 2025-11-20

**Authors:** Xinlan Tang, Yu Liu, Long Chen, Liling Tan, Xiaoyong Wei, Zhijun Chen

**Affiliations:** 1 JiangXi Cancer Hospital, Nanchang, Jiangxi, China; 2 Nanchang University, Nanchang, Jiangxi, China; 3 Department of Nuclear Medicine, The Second Affiliated Hospital of Nanchang University, Nanchang, Jiangxi, China

**Keywords:** hepatocellular carcinoma, extrahepatic metastasis, PD-1 inhibitors, iodine-125 brachytherapy, abscopal effect, immunotherapy

## Abstract

**Introduction:**

Patients with advanced hepatocellular carcinoma (HCC) and extrahepatic metastases face a poor prognosis with limited treatment options.

**Methods:**

We conducted a retrospective study of 84 such patients treated between January 2018 and April 2025 with either PD-1 inhibitor immunotherapy alone or in combination with iodine-125 (^125^I) seed brachytherapy. Propensity score matching (PSM) yielded 25 patient pairs for outcome comparison.

**Results:**

The combination therapy achieved significantly better local tumor control than monotherapy. Progression-free survival was prolonged with the combined treatment (median PFS 22.06 vs. 9 months, *p* = 0.03), although overall survival was not significantly different between the groups. Both treatments markedly reduced serum alpha-fetoprotein levels and alleviated cancer-elated pain, with significant pain score improvements from baseline in each group. No severe treatment-related complications or deaths occurred. Multivariate analysis indicated that adding ^125^I seed implantation was an independent protective factor for disease progression control.

**Discussion:**

In conclusion, combining PD-1 inhibitors with ^125^I seed implantation is a safe and effective strategy to improve local tumor control and progression-free survival in metastatic HCC, although the benefit in overall survival is limited. Further studies are needed to optimize this combined therapy, assess systemic immune effects, and improve long-term outcomes.

**Clinical trial registration:**

https://www.clinicaltrials.gov/, identifier NCT06991907.

## Introduction

Liver cancer is the third leading cause of cancer-related death worldwide, with over one million new cases annually, and a 5-year overall survival (OS) rate of only about 18% (1). Patients with advanced hepatocellular carcinoma (HCC) and extrahepatic metastases have especially poor outcomes and few effective treatments. Surgical intervention often fails to control tumor progression in this setting, and chemotherapy has limited efficacy. Effective therapeutic strategies are scarce, resulting in a 5-year survival rate under 5% for these patients (2).

PD-1 inhibitors have emerged as a cornerstone of immunotherapy for HCC. However, PD-1 inhibitor monotherapy offers limited efficacy, with an objective response rate of only ∼15% in advanced HCC. One major reason is the immunosuppressive tumor microenvironment, which leads to resistance and diminishes the long-term benefits of immunotherapy (3). Meanwhile, iodine-125 (^125^I) seed brachytherapy is a form of internal radiotherapy that continuously emits low-dose γ-rays, inducing DNA double-strand breaks and irreversible damage in tumor cells. This approach is particularly effective against hypoxic, radioresistant cells. The gradual decay of ^125^I seeds allows localized high-dose irradiation to the tumor with minimal exposure to surrounding normal tissues (4). Brachytherapy with ^125^I seeds is a well-established, minimally invasive treatment with few complications and good patient tolerance.

Previous studies have shown that ^125^I seed implantation can achieve significant local tumor control in HCC, and combining brachytherapy with other treatments may improve progression-free survival (PFS) and OS to varying degrees (5, 6). Preclinical research further supports these findings. Animal experiments have demonstrated that combining ^125^I seed radiation with PD-1 blockade significantly inhibits the growth of primary and metastatic tumors (7). Interestingly, this combined approach can even induce an abscopal effect, whereby tumor lesions not directly irradiated also regress. These results suggest a systemic antitumor effect of the combined therapy. Additional preclinical studies indicate that ^125^I brachytherapy can reshape the tumor immune microenvironment and increase immune cell infiltration, thereby enhancing the efficacy of PD-1 immunotherapy (8).

Despite these promising indications, clinical data on combining PD-1 inhibitors with ^125^I seed implantation are lacking. Therefore, we conducted a study to explore the efficacy and safety of this combined treatment in patients with advanced HCC and extrahepatic metastasis. We aimed to determine whether the addition of ^125^I seed brachytherapy to PD-1 immunotherapy could improve tumor control and patient outcomes, ultimately providing a new therapeutic option for this challenging patient population.

## Materials and methods

### Patient selection

We retrospectively collected clinical data for HCC patients treated at Jiangxi Cancer Hospital from January 2018 to April 2025. Eligible patients met the following inclusion criteria: (1) diagnosis of advanced primary HCC according to established criteria (9); (2) at least one measurable extrahepatic metastatic lesion; (3) Eastern Cooperative Oncology Group (ECOG) performance status 0–1 (all included patients were ECOG 0); (4) Barcelona Clinic Liver Cancer (BCLC) stage C; (5) Child-Pugh class A liver function; (6) platelet count > 75 × 10^∧^9/L; (7) life expectancy ≥ 3 months; and (8) complete clinical data available. Exclusion criteria included: (1) inability to adhere to the treatment protocol or cooperate during seed implantation; (2) the presence of a major bronchus or large blood vessel at or near the planned puncture site (posing unacceptable risk for brachytherapy); (3) co-existence of other malignant tumors; (4) severe heart or kidney failure; or (5) incomplete clinical data or loss to follow-up.

### Materials and equipment

All patients underwent ^125^I seed implantation using an 18G Yakko puncture needle and a semi-automatic seed implantation device. The treatment planning system (TPS) was provided by Beijing Tianhang Kelinzhong Technology Co., Ltd. The iodine-125 seeds (from Beijing Atomic Gaoke Biotech Co.) had an activity of 2.22–2.96 × 10^∧^7 Bq each, a half-life of ∼59.6 days, and an effective radiation range of 17–20 mm. All seeds were sterilized by autoclave before use. Serum alpha-fetoprotein (AFP) levels were measured with a Roche Cobas 6000 e602 chemiluminescence analyzer as a tumor marker.

### Treatment procedure

All patients completed their planned treatment and follow-up in the Department of Nuclear Medicine at our hospital. PD-1 inhibitor therapy was administered intravenously at 200 mg every 3 weeks. Patients received one of the following PD-1 inhibitors according to physician discretion: sintilimab, tislelizumab (Tevimbra), or camrelizumab. There was no bias in PD-1 agent usage between the two study groups (see Table 1). Dose reductions or temporary suspensions of the PD-1 inhibitor were allowed based on the severity of any immune-related adverse reactions, following the manufacturers’ guidelines.

**TABLE 1:** Patients’ baseline characteristics before and after propensity score matching (PSM).

	Before PSM (Median, IQR; No., %)			After PSM (Median, IQR; No., %)		
	Group A (*n* = 41)	Group B (*n* = 43)	*p*-value	Group A (*n* = 25)	Group B (*n* = 25)	*p*-value
Gender			0.739			>0.999
Male	37(90.24%)	37(86.05%)		23(92%)	23(92%)	
Female	4(9.86%)	6(13.95%)		2(8%)	2(8%)	
Age			0.370			0.221
Median	61(54.5-66)	55(49-66)		62(55-66)	55(51-66)	
Type of PD-1 inhibitor			0.431			>0.999
Sintilimab	25(60.98%)	23(53.49%)		12(48%)	12(48%)	
Tevimbra	6(14.63%)	6(13.95%)		4(16%)	4(16%)	
Camrelizumab	10(24.39%)	14(32.56%)		9(36%)	9(36%)	
ECOG score			0.534			0.765
0	13(31.71%)	11(25.58%)		9(36%)	8(32%)	
1	28(68.29%)	32(74.42%)		16(64%)	17(68%)	
HBV infection			0.014*			>0.999
Negative	14(34.15%)	5(11.63%)		3(12%)	4(16%)	
Positive	27(65.85%)	38(88.37%)		22(88%)	21(84%)	
AFP (μg/L)			0.513			0.777
<400	22(53.66%)	20(46.51%)		12(48%)	13(52%)	
≥ 400	19(46.34%)	23(53.49%)		13(52%)	12(48%)	
Ascites			0.923			0.508
Absent	29(70.73%)	30(69.77%)		18(72%)	20(80%)	
Mild	12(29.27%)	13(30.23%)		7(28%)	5(20%)	
Tumor size (cm)			0.996			0.773
≤ 3	21(51.22%)	22(51.16%)		13(52%)	14(56%)	
>3	20(48.78%)	21(48.84%)		12(48%)	11(44%)	
Tumor number			0.120			>0.999
1	26(51.22%)	20(46.51%)		14(56%)	14(56%)	
>1	15(48.78%)	23(53.49%)		11(44%)	11(44%)	
Metastasis			0.356			0.774
Lung	26(60.98%)	23(53.49%)		14(56%)	15(60%)	
Bone	15(39.02%)	20(46.51%)		11(44%)	10(40%)	

*Indicates a *p*-value <0.05. Group A: PD-1 inhibitor monotherapy; Group B: PD-1 inhibitor +^125^ I seed brachytherapy. Data are presented as median (IQR) for continuous variables and number (%) for categorical variables. There were no significant differences between Group A and Group B after PSM in age, gender, Type of PD-1 Inhibitor, ECOG score, Child-Pugh class (all patients were Class A), tumor burden, or metastasis sites. ECOG, Eastern Cooperative Oncology Group; HBV, hepatitis B virus; AFP, alpha-fetoprotein; IQR, interquartile range; —, not applicable.

For patients in the combination group, ^125^I seed brachytherapy was performed within 1 week after a PD-1 inhibitor infusion. Target lesions were selected based on clinical significance and accessibility, typically one per patient (or occasionally two in select cases). Priority was given to those that caused significant symptoms (such as giant mediastinal nodal metastases compressing the trachea or great vessels) or those posing a high risk of future complications.

Prior to implantation, all patients underwent routine preoperative evaluations including coagulation profile, liver and kidney function tests, cardiopulmonary assessment, and a local CT scan for treatment planning. An individualized brachytherapy plan was developed collaboratively by nuclear medicine physicians and medical physicists, with a prescribed radiation dose of 80–120 Gy to each target lesion. Depending on lesion location, seeds of appropriate activity were selected to achieve the planned dose: approximately 1.85 × 10^∧^7 Bq seeds for superficial lesions, 3.33 × 10^∧^7 Bq seeds for bone metastases, and 2.22–2.96 × 10^∧^7 Bq for lesions at other sites. Under CT guidance, seeds were implanted into the tumor according to the TPS plan. Immediately after implantation, a CT scan was performed to verify the seed distribution and dosimetry; if under-dosed regions were identified in the target area, additional seeds were placed during the procedure to ensure adequate coverage. For patients whose follow-up imaging showed an insufficient radiation dose to the tumor, a repeat ^125^I seed implantation would be performed following a decision by physicians.

Patients in the monotherapy group (Group A) received PD-1 inhibitor treatment alone without brachytherapy, while the combination group (Group B) received both PD-1 inhibitor therapy and ^125^I seed implantation. All patients continued to receive supportive care and subsequent lines of therapy as needed according to standard clinical practice.

### Data collection and evaluation

Baseline clinical variables recorded for each patient included age, sex, type of PD-1 inhibitor, Child-Pugh liver function class, BCLC stage, ECOG performance status, serum AFP level, and history of hepatitis B virus (HBV) infection. We also documented the number and locations of metastatic tumors, as well as the number and total activity of ^125^I seeds implanted for each patient in Group B. Patients were followed up at 3 and 6 months after the start of treatment, and then every 6 months thereafter. At each follow-up visit, we assessed clinical symptoms [including pain levels using a Numeric Rating Scale (NRS) 0–10], performed laboratory tests (including complete blood counts and liver/kidney function tests), and obtained imaging studies to evaluate tumor size and response. Treatment-related adverse events (TRAEs) were recorded and graded according to the National Cancer Institute Common Terminology Criteria for Adverse Events (CTCAE) version 5.0.

### Tumor response and outcome definitions

Tumor response was evaluated based on changes in the size of target lesions on imaging, in accordance with RECIST 1.1 criteria (10). The longest diameter of each target lesion was measured by two independent radiologists, and their measurements were averaged. If the two measurements differed by more than 1 cm, a third senior radiologist reviewed the images to determine the final measurement. Tumor volume was calculated using the formula for an ellipsoid: volume = π * (a × b × c)/6, where *a*, *b*, and *c* are the orthogonal diameters of the lesion.

For local tumor efficacy assessment of individual lesions, we categorized responses as follows: a lesion was considered Local Effective (LE) if its longest diameter decreased by ≥ 30% (RECIST 1.1). Progressive Disease (PD) for a lesion was defined as an increase of ≥ 20% in longest diameter. Stable Disease (SD) was assigned to any change that did not meet the criteria for LE or PD. Using these lesion-level assessments, we calculated the Local Effective Rate (LER) and Local Control Rate (LCR) for each group: LER = (number of lesions achieving LE/total number of lesions in group) × 100%; LCR = (number of lesions achieving LE or remaining SD/total number of lesions) × 100%. These metrics quantify local tumor control on a per-lesion basis in each treatment group.

We defined local progression-free survival (LPFS) as the time from treatment initiation to progression of the treated (seed-implanted) lesion or to death, and overall PFS as the time to progression at any site or death. Overall survival (OS) was measured from treatment initiation to death from any cause or last follow-up.

### Statistical analysis

Statistical analyses were performed using SPSS version 27.0. Propensity score matching (PSM) was applied to the two treatment groups to balance baseline characteristics and reduce potential selection bias. Continuous variables with approximately normal distribution are reported as mean ± standard deviation and were compared using independent-sample *t*-tests. Continuous data with non-normal distribution are reported as median (interquartile range) and were compared using the Mann-Whitney rank-sum test. Categorical data are presented as number (percentage) and were compared using the chi-square test or Fisher’s exact test, as appropriate. For comparisons of repeated measurements (e.g., tumor size or pain scores over time), a non-parametric Friedman test was used. Survival outcomes (LPFS, PFS, and OS) were analyzed using the Kaplan-Meier method, and differences between groups were assessed with the log-rank test. A two-tailed *P* < 0.05 was considered statistically significant.

## Results

### Baseline patient characteristics

As of April 30, 2025, a total of 84 HCC patients with extrahepatic metastases met the inclusion criteria and were enrolled in this study. Of these, 41 patients received PD-1 inhibitor monotherapy (Group A) and 43 patients received PD-1 inhibitor therapy combined with ^125^I seed implantation (Group B). After 1:1 propensity score matching, 25 matched pairs (50 patients in total) were selected for outcome comparisons. The matching process achieved well-balanced baseline demographics and clinical characteristics between the two groups (Table 1). The median follow-up period for the matched cohort was 11 months (range 4–52 months). There were no significant differences between Group A and Group B after PSM in terms of age, gender, ECOG performance status, Child-Pugh class (all patients were Class A), tumor burden (size and number of metastatic lesions), or sites of metastasis (all *p* > 0.5) (Table 1). Importantly, the types of PD-1 inhibitors used were similar between the two groups. Especially after propensity score matching, the same PD-1 agent was administered to all patients within each matched pair (Table 1).

All patients in Group B underwent successful ^125^I seed implantation procedures. No serious procedure-related complications occurred. In particular, there were no cases of severe hemorrhage, uncontrolled infection, or injury to critical structures during or after the brachytherapy. This excellent safety profile indicates that the ^125^I seed implantation was technically feasible and safe in all treated patients. On average, each Group B patient had 1 (occasionally 2) metastatic lesions treated with seeds. In a few cases where a patient had multiple metastatic sites, only the most significant one or two lesions were implanted, and other metastases in the same patient were left untreated by brachytherapy (managed with systemic therapy alone).

### Short-term efficacy and tumor response

**FIGURE 1:**
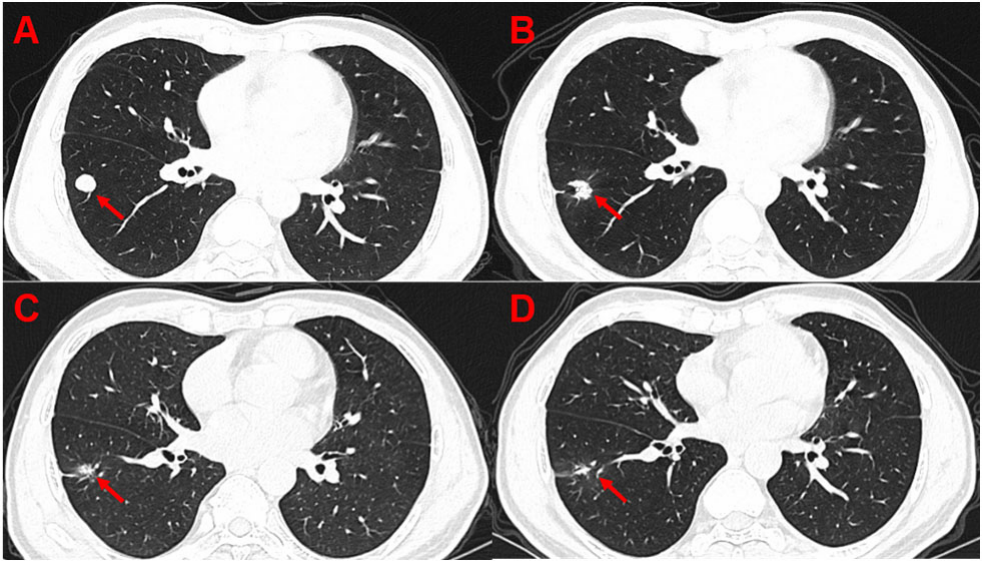
Serial CT images of a representative HCC patient with lung metastases, demonstrating the effect of combined PD-1 inhibitor and ^125^I seed implantation therapy. **(A)** Baseline scan before treatment shows a lung metastatic lesion (arrowed). **(B)** 3 months after combination therapy, the lesion has markedly decreased in size. **(C)** 6 months post-treatment, the lesion is further reduced. **(D)** 12 months post-treatment, the metastatic tumor has shrunk to near-complete resolution. No new lesions or local recurrence were observed at the 12-month follow-up.

We observed clear differences in tumor response between the two treatment groups, particularly at the sites of local therapy. Patients in Group A (PD-1 monotherapy) generally had smaller metastatic tumors at baseline, whereas those in Group B (PD-1 + brachytherapy) had larger median tumor volumes before treatment (median volumes 8 cm^3^ in Group A vs. 44.7 cm^3^ in Group B, *p* = 0.002) (see Table 2). Despite this larger initial tumor burden in Group B, the combination therapy achieved a marked reduction in tumor volume. In Group B, tumor volumes significantly decreased after treatment (*p* = 0.002), whereas in Group A the tumors on average continued to grow during the same period (*p* = 0.041). This finding highlights the potent local tumor control effect provided by ^125^I seed implantation (Figure 1).

**TABLE 2:** Treatment efficacy outcomes and adverse events in Group A vs. Group B.

	Clinical efficacy				Adverse events		
	**Group A (*n* = 25)**	**Group B (*n* = 25)**	***p*-value**		**Group A (*n* = 25)**	**Group B (*n* = 25)**	***p*-value**
**Tumor volume (cm^3^)**				**Fever**			0.061
Before treatment	8(4–20)	44.7(10–278.44)	0.002*	None	18	23	
After 6 months of treatment	10(3.5–36)	12.4(2.05–148.76)	0.027*	Grade 1	2	2	
*p*-value	0.041*	0.002*		Grade 2	5	0	
**AFP (μg/L)**				**Anemia**			0.127
Before treatment	352.2(114.25–980.75)	517(20.99–1242.50)	0.945	None	13	19	
After 6 months of treatment	156.5(93.34–1133)	81(25.1–170.3)	0.004*	Grade 1	7	5	
*p*-value	0.025*	0.004*		Grade 2	5	1	
**NRS**				**Hypothyroidism**			0.082
Before treatment	0(0–1.5)	0(0–4.5)	0.540	None	10	17	
After 6 months of treatment	0(0–0)	0(0–0.5)	0.957	Grade 1	10	7	
*p*-value	0.011*	0.026*		Grade 2	5	1	
				**Fatigue**			0.270
				None	11	15	
				Grade 1	8	8	
				Grade 2	2	2	
				**Leukopenia**			0.130
				None	10	15	
				Grade 1	6	7	
				Grade 2	9	3	

*Indicates a *p*-value <0.05. Clinical efficacy metrics include tumor volume (median cm^3^) before treatment and at 6 months post-treatment, serum AFP levels (median μg/L) before and after 6 months, objective response rate (ORR) and disease control rate (DCR) by RECIST 1.1, and pain score improvements. CR, complete response; PR, partial response; SD, stable disease; PD, progressive disease; ORR, CR + PR; DCR = CR + PR + SD; NRS, Numeric Rating Scale for pain.

According to RECIST 1.1 imaging criteria, more patients in Group B achieved a significant tumor size reduction than in Group A at each follow-up interval (3 months, 6 months, and at the end of the study). Specifically, looking at individual lesions, the combination therapy group showed higher response rates despite starting with larger tumors. Correspondingly, the LER and LCR were significantly higher in Group B compared to Group A at 3 months, 6 months, and at final assessment (all *p* < 0.05; Table 3). Figure 1 illustrates a representative case of a patient with pulmonary metastases who received the combined treatment, demonstrating progressive shrinkage of the lesions over time and nearly complete resolution of visible tumor by 12 months, with no local recurrence.

**TABLE 3:** Local tumor response and control rates at 3 months, 6 months, and study endpoint in Group A vs. Group B.

Follow-up time	LE	SD	PD	*p*-value	LER (%)	LCR (%)
3 months				0.002*		
Group A	4	13	8		16%	68%
Group B	14	10	1		56%	96%
6 months				0.010*		
Group A	4	11	10		16%	60%
Group B	13	9	3		52%	88%
End of study				0.035*		
Group A	3	4	18		12%	28%
Group B	11	3	11		44%	56%

*Indicates a *p*-value <0.05. LE = number of lesions with local effective response (≥ 30% tumor reduction); SD = number of lesions with stable disease; PD = number of lesions with progressive disease. LER = local effective rate = (LE/total lesions) × 100%. LCR = local control rate = ((LE + SD)/total lesions) × 100%.

Beyond local lesion response, the combination therapy also translated into better systemic tumor control. Baseline AFP was similar between the two groups (median ∼300–500 μg/L, *p* > 0.5, Table 2). After 6 months of therapy, AFP levels had decreased significantly in both groups, but the reduction was much greater in the combination group. The median AFP at 6 months dropped to 81 μg/L in Group B, compared to 156.5 μg/L in Group A (*p* = 0.004, Table 2). These AFP results indicate that overall tumor burden was reduced more effectively with PD-1 + ^125^I therapy than with PD-1 alone, aligning with the imaging findings.

**FIGURE 2:**
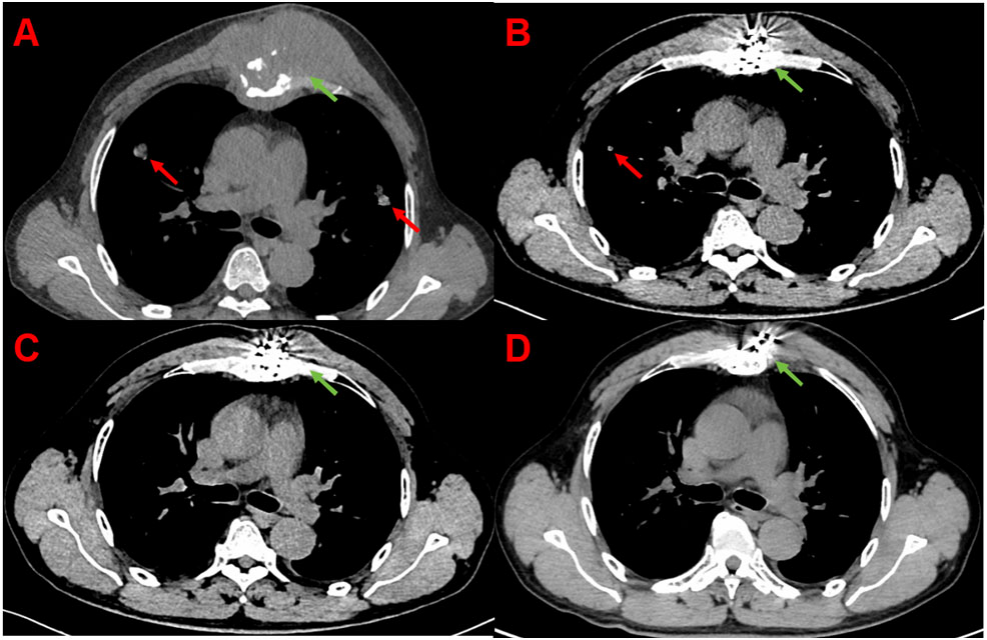
Serial CT images of a representative HCC patient with a thoracic vertebral bone metastasis, before and after combination therapy. **(A)** Baseline image shows a destructive metastasis in a thoracic vertebra (arrow). **(B)** 3 months after PD-1 inhibitor plus ^125^I seed implantation into the lesion, the metastatic tumor has regressed significantly. **(C)** 6 months post-treatment, only a small residual lesion remains. **(D)** 12 months post-treatment, the treated bone bone metastasis showed almost complete resolution with no evidence of recurrence. The patient experienced substantial pain relief and no neurological complications following treatment.

Notably, in Group B we observed that some metastatic lesions not directly implanted with seeds also showed regression or prolonged stability, whereas in Group A (PD-1 alone) the non-target lesions more often progressed or showed only transient stabilization. This observation suggests the presence of a systemic or abscopal effect induced by the synergy of radiotherapy and immunotherapy. For instance, one patient in Group B had two lung metastatic lesions at baseline, but only the larger lesion was treated with ^125^I seeds. Remarkably, at the 6-month follow-up, the untreated smaller lung lesion had also decreased in size despite not receiving local radiation. We present this case in Figure 2. Figure 2A shows the baseline CT with two lung lesions (the green arrow indicates the lesion that was implanted with seeds, and the red arrow points to a second lesion that was left untreated by brachytherapy). Figure 2B displays the 6-month follow-up CT, where both lesions have regressed—the treated lesion shrank dramatically, and the untreated lesion also became smaller. Such systemic tumor responses were rarely seen in the monotherapy group. We reference this example to highlight that the combination therapy may trigger immune-mediated tumor suppression beyond the irradiated sites. (Further analysis of this phenomenon is provided in the Discussion.)

### Symptomatic relief (pain control)

Effective palliation of cancer pain is an important outcome for patients with metastatic HCC, especially those with bone metastases. In this study, a significant proportion of patients had bone metastases (approximately 40–46% in each group; see Table 1), many of whom experienced tumor-related pain at baseline. We evaluated patient-reported pain using the Numeric Rating Scale (NRS, 0–10) in both groups. Baseline pain scores were moderate on average (*p* > 0.5). After treatment, both groups experienced significant pain relief compared to their baseline. By 3 months, many patients reported a noticeable reduction in pain intensity, and by 6 months the median pain scores in both groups had decreased to 0 (IQR 0–0.5 in Group B, and 0–0 in Group A; see Table 2). This reflects that disease control was accompanied by substantial alleviation of cancer-related pain over time.

While the degree of pain improvement was comparable between the groups quantitatively (both showed statistically significant reductions in NRS from baseline, and there was no significant difference in the magnitude of pain score change between Group A and Group B, *p* > 0.05), we did observe some qualitative differences. Patients in the combination therapy group often experienced faster and more complete pain alleviation, especially those with painful bone lesions that were directly treated with ^125^I seeds. In Group B, some patients reported marked pain relief within weeks after seed implantation, and a few had complete resolution of pain symptoms by the 6-month follow-up. In contrast, pain reduction in Group A, while present, tended to be more gradual. These clinical observations suggest that brachytherapy contributed to rapid pain palliation, likely by locally controlling tumor growth in bones or other structures causing pain.

By 6 months, however, both groups had achieved low absolute pain scores, indicating that with ongoing systemic therapy, pain can be managed in advanced HCC patients. We interpret these results cautiously, considering pain control as a secondary outcome. The improvement in pain is likely a consequence of successful tumor control, rather than a direct analgesic effect of the treatments. Nonetheless, the data confirm that the combined modality did not compromise pain management—if anything, it enhanced early pain relief for those with localized painful lesions—and overall, patients in both groups saw meaningful improvement in quality-of-life related to pain.

### Survival outcomes

**FIGURE 3:**
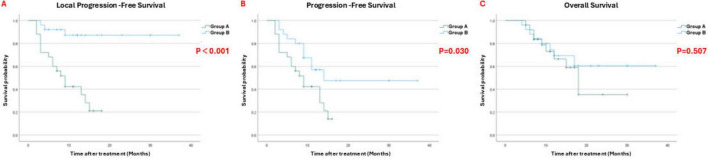
**(A)** Kaplan-Meier curves for local progression-free survival (LPFS), **(B)** progression-free survival (PFS), and **(C)** overall survival (OS) in the two treatment groups after propensity score matching.

Kaplan-Meier survival analyses were performed to evaluate the impact of the treatments on time-to-event outcomes (Figure 3). After PSM to balance baseline tumor burden and other factors, the combination group showed significantly prolonged LPFS compared to the monotherapy group. The median LPFS was 32.96 months (95% CI: 28.69–37.24) in Group B vs. 9.0 months (95% CI: 6.09–11.91) in Group A (*p* < 0.001) (Figure 3A). Similarly, PFS was longer in Group B: the combination therapy yielded a median PFS of 22.06 months vs. 9.0 months with immunotherapy alone. The PFS curves diverged in favor of Group B (Figure 3B). These results demonstrate that the addition of ^125^I brachytherapy provided a significant benefit in delaying tumor progression.

In contrast, OS did not differ significantly between the two groups in our study, despite the PFS benefit. The median OS was around 26.34 months in Group B and 18.00 months in Group A, and this difference was not statistically significant (*p* = 0.507). The OS curves (Figure 3C) overlap considerably. Several Group B patients ultimately succumbed to disease progression in sites that were not amenable to local therapy, which may explain why the significant PFS improvement did not translate into a longer OS within the follow-up period. We note that follow-up is ongoing for some patients, but at the current median follow-up of 23 months, a definitive OS benefit has not emerged for the combination. In addition, our multivariate analysis of all factors potentially affecting patients’ LPFS and OS showed that combination therapy is a protective factor for liver cancer patients with extrahepatic metastasis (Table 4).

**TABLE 4:** Univariate and multivariate Cox regression analysis for local progression-free survival (LPFS) and overall PFS after PSM.

	LPFS						PFS					
Variables	Univariate analysis			Multivariate analysis			Univariate analysis			Multivariate analysis		
	HR	95%CI	*p*-value	HR	95%CI	*p*-value	HR	95%CI	*p*-value	HR	95%CI	*p*-value
**Age**												
Median	0.974	0.932–1.019	0.253				0.972	0.936–1.008	0.123			
**Treatment method**												
PD1 inhibitor	Ref			Ref			Ref			Ref		
PD1 + ^125^I brachtherapy	7.349	2.148–25.139	0.001*	18.91	4.47–80.01	<0.001*	2.185	1.029–4.641	0.042*	4.779	1.908–11.97	<0.001*
**Gender**												
Female	Ref						Ref					
Male	24.157	0.44–13349.9	0.323				1.177	0.278–4.989	0.825			
**ECOG score**												
0	Ref						Ref					
1	1.132	0.451–2.842	0.791				0.952	0.449–2.020	0.899			
**HBV infection**												
Negative	Ref						Ref					
Positive	1.018	0.297–3.489	0.978				0.572	0.232–1.414	0.227			
**AFP (μg/L)**												
<400	Ref			Ref			Ref			Ref		
≥ 400	4.580	1.619–12.957	0.004*	3.079	0.77–12.37	0.113	2.593	1.203–5.588	0.015*	1.074	0.738–3.413	0.903
**Ascites**												
Absent	Ref			Ref			Ref			Ref		
Mild	3.394	1.275–9.033	0.014*	6.78	1.40–32.69	0.017*	4.043	1.760–9.290	<0.001*	6.228	1.69–22.95	0.006*
**Tumor size (cm)**												
≤ 3	Ref			Ref			Ref			Ref		
>3	2.692	1.012–7.162	0.047*	1.413	0.43–4.65	0.570	4.3	1.8–10.182	<0.001*	2.066	0.747–5.713	0.162
**Tumor number**												
1	Ref						Ref			Ref		
>1	2.423	0.914–6.423	0.075				2.435	1.081–5.483	0.032*	1.685	0.649–4.37	0.284
**Metastasis**												
Lung	Ref			Ref			Ref			Ref		
Bone	2.944	1.163–7.449	0.023*	4.025	1.16–13.99	0.028*	4.607	1.914–11.09	<0.001*	5.732	2.0–16.429	0.001*

*Indicates a *p*-value <0.05. HR, hazard ratio; CI, confidence interval; Ref, reference category for HR; —, not significant.

### Adverse events and safety

Both treatments were generally well-tolerated. No treatment-related deaths occurred. Immune-related adverse events (irAEs) associated with PD-1 inhibitor therapy were observed in both groups, but mostly at low grades (Grade 1–2) and manageable. The most common adverse events were mild fatigue, fever, rash or pruritus (immune-related skin reactions), transient liver enzyme elevation, and hypothyroidism, all known side effects of PD-1 antibodies. There were no Grade ≥ 3 toxicities attributed to the PD-1 inhibitors in either group. Importantly, we did not observe any additional toxicity from the ^125^I brachytherapy. In Group B, there were no acute complications such as seed migration, radiation-induced damage to adjacent organs, or severe bleeding/infection from the implantation procedure. Also, we did not find evidence of exacerbated systemic side effects in Group B patients; for instance, the rates of fever, cytopenias, or fatigue were similar between the two groups (Table 2 shows that none of the differences in specific adverse event incidence reached statistical significance, all *p* > 0.05). This suggests that combining local radiotherapy with immunotherapy did not synergistically increase toxicity. Hematologic profiles were monitored, and while some patients experienced mild leukopenia or anemia during therapy, these were attributed to PD-1 therapy or underlying disease and occurred at similar frequencies in both arms (Table 2). Notably, we did not see severe lymphopenia in Group B that could be linked to radiation—possibly due to the localized nature of brachytherapy and the relatively limited radiation field, which spares the majority of circulating lymphocytes.

In summary, the safety analysis indicates that the combined modality treatment is feasible and does not pose undue risk to patients beyond the known side effects of immunotherapy. All patients in Group B successfully completed brachytherapy and continued PD-1 inhibitor treatments, with no discontinuations due to adverse events.

## Discussion

This study demonstrates that combining PD-1 inhibitor immunotherapy with ^125^I seed brachytherapy can improve clinical outcomes for patients with advanced HCC and extrahepatic metastases, compared to immunotherapy alone. The most striking benefit of the combined treatment was in local tumor control, which is reflected in a dramatically higher LPFS and LCR. Effective local therapy in metastatic sites contributed to slowing overall disease progression, as evidenced by the prolonged PFS in the combination group. These results align with the rationale that controlling key sites of disease can prevent complications and subsequent spread, thereby delaying progression.

Notably, some patients in the combination group experienced regression of metastatic lesions that were not directly implanted with seeds, whereas in the monotherapy group the non-target lesions often continued to progress or only transiently stabilized. This observation suggests the presence of an abscopal effect induced by the synergy of radiotherapy and immunotherapy. The abscopal effect—wherein localized radiation treatment leads to regression of tumor lesions outside the radiation field—has been documented in the context of radiotherapy combined with immune checkpoint blockade (8). The combination of ^125^I brachytherapy with PD-1 inhibition in our study appeared to stimulate a systemic anti-tumor immune response that helped control distant tumor sites in addition to the directly treated lesions (as illustrated in Figure 2). While our study was not designed to formally prove an abscopal effect, these anecdotal results are encouraging and consistent with prior reports and preclinical models (5–8).

The trends in serum AFP levels further support the enhanced efficacy of the combined treatment beyond local effects. We observed that after 6 months, AFP levels were significantly lower in the combination group than in the monotherapy group, indicating a greater reduction in overall tumor burden with combined therapy. This aligns with our imaging results and suggests that the benefits of combination therapy were not confined to the seeded lesions, but extended to tumor control throughout the body. In other words, the addition of radiotherapy likely potentiated the systemic impact of immunotherapy, resulting in more tumors responding (either directly or indirectly via immune-mediated mechanisms).

The synergy between ^12^5I brachytherapy and PD-1 immunotherapy likely arises from their complementary mechanisms of action. Continuous low-dose radiation from ^125^I seeds can act as an *in situ* tumor vaccine—a concept supported by preclinical studies (8). Radiation induces immunogenic tumor cell death, leading to the release of tumor antigens and pro-inflammatory signals. This can promote dendritic cell maturation and antigen presentation to T cells. At the same time, PD-1 inhibitors unleash the adaptive immune response by blocking the PD-1/PD-L1 pathway, thereby restoring T-cell activity against tumor cells. The combination of localized radiation with checkpoint blockade can convert the irradiated tumor into a nidus of immune activation. Additionally, ^125^I brachytherapy may favorably alter the tumor microenvironment to further potentiate immunotherapy. Radiation has been reported to reduce populations of immunosuppressive cells such as myeloid-derived suppressor cells and to increase infiltration of cytotoxic T lymphocytes; this creates a positive feedback loop between radiotherapy and immunity, sometimes referred to as the “radioimmune” effect. Li et al. (11) described this synergistic anti-tumor interaction in which ^125^I-induced abscopal effects amplified the efficacy of PD-1/PD-L1 blockade in a preclinical model. Our clinical outcomes are in line with this concept, as the combination therapy yielded systemic benefits beyond the local radiation field.

Furthermore, the sequencing of radiotherapy and immunotherapy in our regimen may have contributed to the observed efficacy. We intentionally performed brachytherapy shortly after administering the PD-1 inhibitor (within 1 week of infusion) to maximize their cooperative effect. This timing was intended to ensure that activated T cells (stimulated by the PD-1 blockade) would be in circulation and could home to tumor sites at the time of radiation-induced tumor antigen release. By delivering radiation when the immune system is already “primed” and not deeply immunosuppressed, we likely enhanced the probability of an effective anti-tumor immune response. Equally important, using localized brachytherapy helped minimize damage to circulating lymphocytes and lymphoid organs. Radiotherapy is known to sometimes cause lymphopenia (radiation can deplete lymphocytes, especially if large blood volumes or lymphoid regions are irradiated), which can counteract the benefits of immunotherapy. In our approach, the ^125^I seeds deliver radiation directly to the tumor with rapid dose fall-off, sparing most normal tissues. This means lymphatic drainage areas and the systemic immune cell pool were largely preserved, reducing the risk of radiation-induced systemic immunosuppression. We hypothesize that this combination of proper timing and focused radiation allowed us to harness the immunostimulatory effects of radiation (and achieve abscopal responses in some cases) while avoiding significant detrimental effects on the immune system’s overall function. This aspect underlines a crucial point for combined modality therapies: treatment scheduling and modality selection (e.g., brachytherapy vs. external beam) should be optimized to maintain immune competence during therapy.

Aside from tumor control, palliation of symptoms is a key goal in metastatic HCC care. Many patients in our study had painful bone metastases or other cancer-related pain. Our results indicate that both treatment strategies provided meaningful pain relief, reflecting the benefit of tumor control on symptom burden. The use of ^125^I seed implantation in Group B likely contributed to rapid pain relief for bony lesions, through multiple mechanisms. By killing tumor cells *in situ*, brachytherapy reduces the production and release of pain-inducing substances (such as bradykinin, prostaglandins, and inflammatory cytokines) from the tumor. Radiation-induced tumor shrinkage can relieve pressure on bones, nerves, or other structures, thereby reducing nociceptive stimuli. Additionally, continuous low-dose irradiation may induce fibrosis and microthrombosis in the tumor’s blood vessels, reducing tumor perfusion and the leakage of irritating factors that trigger pain.

These mechanisms are supported by prior research on brachytherapy for bone metastases, which has shown effective pain control and even complete pain responses in a substantial proportion of patients (12). In our study, both monotherapy and combination therapy led to statistically significant reductions in pain scores after 6 months of treatment compared to baseline, underscoring that controlling tumor activity (via systemic therapy, local therapy, or both) can alleviate cancer pain. We observed that the combination therapy group tended to experience faster and more complete pain alleviation in clinical practice, although quantitatively the pain score improvements were similar between groups by 6 months. We interpret this to mean that brachytherapy offers an added benefit in the speed of pain palliation for appropriately selected patients (especially those with localized pain from metastases), even though ultimately both groups can achieve pain control with ongoing therapy. It is important to note that pain relief was a secondary outcome in this study; thus, while the improvements are encouraging and contribute to quality of life, our trial was not primarily designed to compare analgesic effects between treatments. Nevertheless, our findings concur that ^125^I seed implantation is a valuable modality to alleviate cancer pain in metastatic HCC, improving patient comfort and daily functioning as part of a multimodal approach.

Safety is a critical consideration when introducing any new combined-modality treatment. In the present study, the combination of PD-1 immunotherapy with ^125^I seed brachytherapy was well tolerated. The adverse events observed were mainly those expected from PD-1 inhibitor therapy (immune-related reactions such as fever, fatigue, dermatologic effects, endocrine disturbances, etc.), and these were mostly low-grade (Grade 1–2) and manageable with standard supportive care. Importantly, the brachytherapy procedure did not result in additional severe toxicity. We did not encounter any acute complications attributable to seed implantation, such as hemorrhage or infection, and no delayed complications like radiation damage to organs or seed migration were noted on follow-up. Past analyses have indicated that combining loco-regional therapies with systemic treatments can sometimes increase toxicity, but in our experience we did not observe any synergistic adverse effects. No life-threatening events occurred in either group, and notably no patient had to discontinue treatment due to toxicity. This safety profile aligns with the notion that ^125^I brachytherapy is a localized treatment with limited systemic side effects, making it a safe adjunct to systemic immunotherapy. Our results reinforce that with careful patient selection and adherence to proper procedural protocols, ^125^I seed implantation can be performed without major risk even in advanced cancer patients. These findings are reassuring for future attempts to integrate brachytherapy with immunotherapy in clinical practice.

In comparing our survival outcomes to historical data, it is notable that even with advanced disease, the median OS in both groups of our study (approximately 18 months) was longer than the 4–9 months reported in older studies for advanced HCC with extrahepatic spread (2). This improvement likely reflects the advances in therapy over the past decade, including the use of modern immunotherapies and better supportive care. However, we did not find a statistically significant difference in OS between the combination and monotherapy arms. This contrasts with some prior studies in other contexts; for instance, a meta-analysis by Zhu et al. (6) found that adding ^125^I brachytherapy to transarterial chemoembolization (TACE) significantly improved 1-year OS in HCC compared to TACE alone.

In our immunotherapy context, the lack of an observed OS benefit could be due to several factors. We hypothesize that the extremely advanced stage and heavy tumor load in our patient population limited the impact of local therapy on overall survival. Many patients had multiple metastatic sites and aggressive disease. Even though combination therapy controlled some lesions effectively, these patients likely succumbed to progression in other sites or to decline in liver function and performance status (factors unrelated to the treated lesions *per se*). End-stage HCC often involves a vicious cycle of immunosuppression and multi-organ dysfunction that may not be fully overcome by controlling a subset of tumors. It is possible that if combination therapy were applied earlier in the disease course—when tumor burden is lower and patients have better physiological reserves—it might translate into an OS advantage. Our data suggest that timing is critical; intervention with radiotherapy + immunotherapy should ideally occur before the patient reaches end-stage immunosuppression and organ failure, in order to capture the maximum benefit on survival.

This study has several limitations. First, it is a single-center retrospective analysis with a relatively small sample size. Although we employed PSM to balance known confounders, the retrospective design is subject to potential selection biases and unmeasured confounding factors. Second, the follow-up duration (median ∼23 months after PSM) may be insufficient to fully assess long-term survival benefits or late toxicities; a longer observation period would provide more insight into the durability of responses and any delayed effects of therapy. Third, while our findings are promising, they need to be validated in a broader population. Future research should include prospective, multi-center trials with larger cohorts to confirm the efficacy and safety of the PD-1 inhibitor plus ^125^I seed combination. Randomized controlled trials would be especially valuable to eliminate bias and definitively determine any improvement in overall survival.

Biomarker studies should also be integrated to help identify which patients are most likely to benefit from this combined modality—for example, evaluating tumor PD-L1 expression, immune cell infiltration in the tumor microenvironment, or specific genetic markers. In addition, we acknowledge that we did not analyze certain blood-derived inflammatory or immune markers (such as the neutrophil-to-lymphocyte ratio, NLR) in this study. Incorporating such markers could provide a better understanding of the systemic immune status of patients and how it correlates with response to therapy; future studies should consider collecting and analyzing NLR or other immune parameters as part of the assessment. Lastly, quality-of-life outcomes should be rigorously assessed in future studies, since pain relief, performance status, and other patient-reported outcomes are important considerations for advanced cancer therapies and may not be fully captured by traditional tumor metrics.

Another clinically relevant factor is the impact of underlying hepatitis B virus (HBV) infection. Approximately 77% of our cohort were HBV-positive, and chronic HBV may alter the immune microenvironment via mechanisms like T-cell exhaustion and persistent hepatic inflammation, potentially influencing influencing immunotherapeutic efficacy. After PSM, no significant difference in HBV positivity distribution was observed between groups, mitigating potential confounding by HBV status. However, future studies should explore how HBV status affects disease progression and outcomes in advanced HCC, as viral etiology-related differential treatment responses may emerge.

In summary, our results indicate that PD-1 inhibitor immunotherapy combined with ^125^I seed implantation is an effective treatment approach for advanced HCC with extrahepatic metastasis. The combination therapy achieved superior control of local tumor lesions, significantly prolonged progression-free survival, and provided meaningful pain relief, all while maintaining an acceptable safety profile. However, the addition of ^125^I brachytherapy did not significantly extend overall survival in this high-risk cohort, likely due to the aggressive nature of widespread metastatic disease. Further optimization of the treatment strategy—such as integrating other systemic therapies, selecting appropriate patients, or intervening at an earlier stage—may be required to improve long-term survival. This combined modality represents a promising avenue to enhance the therapeutic outcomes for metastatic HCC, and it warrants continued investigation in prospective clinical trials.

## Data Availability

The raw data supporting the conclusions of this article will be made available by the authors, without undue reservation.
